# Aftershock Production in Mean Field Avalanche Models with Static Fields

**DOI:** 10.3390/e28091003

**Published:** 2026-09-08

**Authors:** Jordi Baró

**Affiliations:** Departament de Física de la Matèria Condensada, Universitat de Barcelona, 08028 Barcelona, Spain; jbaro@ub.edu

**Keywords:** aftershocks, avalanches, mean-field theory, stochastic process, branching process, self-excitation, epidemic growth

## Abstract

Advanced seismic hazard assessment frameworks rely on stochastic models which include aftershock production in the form of branching or self-exciting point processes. Such empirical constructs are based on debated statistical laws observed across catalogs of natural seismicity, which lack a derivation from first principles. Here, we derive the statistics of aftershock production in a generalized mean-field model of avalanche dynamics with static random thresholds and bimodal relaxation. The number of direct aftershocks is statistically characterized as a renewal counting process accounting for Borel-distributed refractory intervals. At the large-number limit, the expected number of aftershocks is proportional to the size of the parent event with a characteristic scale linearly depending only on the branching parameter governing refractory intervals, whereas the variance follows a distinct parabolic dependence with the same parameter. This model provides a rationale for the overdispersion in aftershock production observed in field data with respect to the Poissonian offspring numbers of standard Hawkes models, but it cannot explain the ubiquity of self-similar aftershock production found in catalogs and lab experiments.

## 1. Introduction

Aftershock production is a source of spatiotemporal clustering first described in seismology and observed across a broad class of systems exhibiting intermittent “crackling-noise” dynamics [1,2]. These include a broad variety of acoustic emission experiments in rock fracture [3,4,5,6], crack propagation [7], granular shearing [8], or the external stressing of silica glasses [9,10,11], wood [12,13] or charcoal [14,15], and even structural phase transitions [16]. Within a point process framework, we define aftershocks as point events caused by transient increases in the activity rate triggered by preceding events, implying history dependence, rather than by variations in the exogenous driving. When such an increase can be traced to one specific event, we can establish direct causal links between the triggered aftershocks and their parent event. We define this phenomena as inter-event triggering, which can be described in terms of stochastic branching processes. Epidemic-type aftershock sequence (ETAS) models designate a category of branching processes combining the activity caused by both an exogenous driving and inter-event triggering, specifically incorporating the common statistical laws observed in seismic catalogs [17]. In the point process framework, ETAS models are often interpreted as self-exciting, a.k.a. Hawkes, point processes [18], where the history-dependent expected rate of events, a.k.a. the intensity (λ(r,t)), is a linear superposition of a Poisson background rate $\lambda_{0}\left(r,t\right)$, initiating independent triggering trees, and the excitation generated by each previous event *i*:

(1) $$\lambda \left(r,t\right)=\lambda_{0}\left(r,t\right)+\sum_{i}\Psi_{i}\left(r-r_{i},t-t_{i},S_{i}\right)$$

This excitation term ($\Psi_{i}$) is modeled as the product of a normalized kernel describing the spatiotemporal probability distribution [19,20] and scaled by a productivity term, determining the expected number of direct aftershocks generated by an event of size $S_{i}$ in generic magnitude (e.g., energy or moment):

(2) $$n\left(S_{i}\right)=KS_{i}^{\alpha },$$

where K=n(1).

To date, ETAS models remain largely empirical, being grounded on field and experimental observations, but lacking a formal derivation from physical principles. This empirical nature has motivated the continued examination of both the ETAS formulation and its parameter estimation procedures. For instance, estimates of aftershock productivity are known to be biased at short times by the transient increase in the magnitude of completeness following large earthquakes, motivating the development of modified ETAS formulations that explicitly account for catalog incompleteness [21,22,23]. Here, we focus on the so-called productivity law [19,24], which is linked to the size scaling in the production term presented in Equation (2). While beyond additional geomechanical dependencies [25], the existence of an average scaling relation between size (or magnitude) and productivity seems intuitive, the precise statistical form and parameter values of aftershock production remain disputed.

A first debated issue concerns the apparent scale-free nature of aftershock production. In ETAS models, the value of the productivity exponent α depends on the definition of size, e.g., seismic moment, radiated energy, or rupture area, which are typically related by power laws. The standard ETAS model assumes the Gutenberg–Richter law [26] translated in sizes (*S*) as a scale-free distribution:

(3) $$N\left(S_{i}>S\right)∼S^{-b}.$$

When expressed in consistent size units, both exponents α and *b* define a scale-invariant ratio α/b which plays a central role in controlling the statistics and topology of the resulting triggering trees [27,28]. (Both power-law expressions (Equations (2) and (3)) take exponential forms when working with logarithmic magnitude measures. In particular, for earthquake magnitudes, α≈b≈1). In such an idealized scale-free limit, ETAS models typically consider α/b≤1 such that branching ratios can be constrained to an average branching parameter 〈n(S)〉≤1 [27] with the particular case α=b corresponding to the self-similar branching aftershock sequence (BASS) model [29]. Several studies have reported catalog statistics consistent with a near-critical branching regime and the self-similar BASS limit [29,30,31]. Similar self-similar values are observed in acoustic emission catalogs obtained during the compression of highly heterogeneous materials exhibiting mean-field exponents, such as porous glasses [9] and wood [12], the crackling of ethanol-dampened charcoal due to evaporation [14], also displaying mean-field exponents, and single crack propagation close to the depinning limit [7]. Tectonophysical models reproduce the scale-free limit for quasi 2D systems, i.e., for large events, by imposing a linear relation between aftershock production and Coulomb stress transfer [32,33]. This explanation cannot account for the coincidence in the aforementioned lab experiments, which suggest a more universal origin.

A second open issue concerns the statistical fluctuations around the mean productivity law in Equation (2). Simple self-exciting processes assume that the number of triggered events follows a Poisson distribution, whereas field observations have reported overdispersion, i.e., fluctuations exceeding Poissonian expectations, with offspring numbers better described by negative-binomial, exponential or geometric distributions [34,35,36,37]. Regional and temporal heterogeneity in the geomechanical properties of seismogenic systems is known to affect aftershock productivity and can contribute to the apparent overdispersion of offspring statistics [25]. In addition, as explored in the current work, overdispersion might be intrinsic to aftershock production and appear as well in homogeneous settings. Rather than proposing a closed answer to these debates, here, we examine the expected behavior of aftershock production from first principles considering a generalized mean-field model.

At the micromechanical level, aftershock production is studied within the framework of avalanche dynamics, describing the fast transformation events, recorded as earthquakes or acoustic emission signals, as cascading processes, a.k.a., *avalanches*, emerging from the interplay between positive feedback interactions and disorder fields. Several works have highlighted the role of secondary relaxation time scales, slower than the immediate static-stress transfer, to the production of aftershocks. Temporal and spatiotemporal clustering is achieved when including such slow mechanisms of energy transfer in the form of damage rheology [38], viscoelastic relaxation [39,40,41,42,43,44,45,46], pore-pressure diffusion [47,48,49], or rate-and-state friction [40,50,51], operating alongside fast elastic and elasto-plastic interactions. While sudden transformation events identified as dislocation motion, frictional slip, or fracture are directly detectable by seismic or acoustic remote sensing [9,41,45,48,52,53], such slow relaxation processes are harder to detect, yet they are still able to slowly drive the system toward new metastable states, eventually triggering the secondary fast events that we identify as aftershocks.

In Section 2, we adopt a generalized description of avalanche dynamics considering mean-field interactions and a discrete field of random disorder [54]. The bimodal mean-field (BMF) model introduced here switches the instantaneous relaxation assumed in Baró and Corral [54] by a *bimodal* relaxation kernel with two distinct temporal scales, both faster than driving, akin to the quasistatic transient hardening considered by Baró and Davidsen [45], Dahmen et al. [55]. This separation of time scales naturally leads to the stochastic processes described in Section 3. The events that, in the absence of slow relaxation mechanisms, are identified as independent avalanches decompose into smaller temporally correlated *sub-avalanche* events forming aftershock sequences. Previous works considering slow time scales also reported similar fragmentation mechanics [40,41,45,55]. The resulting stochastic process can be interpreted as a nested superposition of two branching process: (i) the fast cascading events building sub-avalanches and represented as point events in the ETAS formulation, which maps exactly into a Poisson branching process as in Baró and Corral [54], and (ii) the triggering causal links between such sub-avalanche events leading to aftershock production in terms of the ETAS formulation. Instead of the Poisson number expected in self-exciting processes and assumed in the standard ETAS model, the number of aftershocks directly triggered by a parent event can be mapped into a renewal counting process consisting of random times plus independent refractory times whose distribution inherits the statistics of the branching process underlying the sub-avalanche propagation. In Section 4, we derive the expected number and variance of aftershocks under a renewal-counting-process central-limit (CLT) theorem, i.e., considering a large number approximation, and the productivity law in the terms presented in Equation (2), separating the statistics for first-generation aftershocks and higher generations. In Section 5, we discuss the observable aftershock production in terms of the ETAS formulation and field studies.

## 2. The Mean-Field Aftershock Model

The proposed micromechanical model of aftershock production inherits the formalism of the stochastic mean-field model proposed by Baró and Corral [54] and generalizes the mechanisms for aftershock production introduced by Baró and Davidsen [45]. Let us consider a scalar mean field (ξ∈R) slowly driven by a monotonously growing external field B(t) across a disorder field, consisting of a discrete set of independent and identically distributed (i.i.d.) random thresholds $\left\{X_{1},\;X_{2}>X_{1},\;\ldots ,\;X_{i}>X_{i-1},\;\ldots \right\}$. Let us consider that the dynamical evolution of ξ(t) takes the form of the linear superposition:

(4) $$\xi \left(t\right):=B\left(t\right)+\sum_{i|X_{i}<\xi \left(t\right)}R_{i}\left(t-t_{i}\right),$$

where the *internal term* $R_{i}\left(\tau \right)$ is a positive monotonic response caused by the activation of a threshold $X_{i}<\xi$ such that $R_{i}\left(\tau <0\right)=0$ and $R_{i}\left(\tau \to \infty \right)=l_{i}$ and $l_{i}$ is a finite long-term field increase or interaction strength. The value $t_{i}$ marks the activation time of each threshold such that $\xi \left(t\to t_{i}^{-}\right)-X_{i}$ turns positive. A last important measure is the threshold density, ν(ξ), which is well defined for independent $\left\{X_{i}\right\}$ as the expected number of thresholds $\left\{X_{i}\right\}\in \left(\xi ,\xi +d\xi \right)$ for units of δξ.

This mean-field model represents the avalanche dynamics of a broad category of interactive mean-field models with uncorrelated disorder within quasistatic and athermal conditions [54]. As an example, this construction is isomorphic to the random field Ising model (RFIM) with mean-field interactions, where the threshold set corresponds to the internal random fields $\left\{h_{i}\right\}$ and the mean-field can be rewritten in terms of the reduced magnetization, i.e., the normalized sum of spins $m\left(t\right):=\sum S_{i}/N$, as: ξ(t):=H(t)+Jm(t), where the adimensional terms H(t) and *J* are the external magnetic field and magnetic exchange term, respectively, and the threshold density ν is inversely related to a local measure of disorder. The same Equation (4) reproduces the avalanche dynamics in the democratic fiber bundle model, and it is a good approximation of the self-organized critical (SOC) slip mean-field theory, which was introduced as a conceptual avalanche model for earthquakes [55]. At the thermodynamic limit, those models differ only in the evolution of the model parameters and not in the local mechanics and statistics of avalanches [54].

### 2.1. Avalanches Modeled as Unimodal Relaxation

Avalanches emerge as instable intervals of the the mean-field in Equation (4) due to the implicit relation between ξ(t) and $\left\{R_{j}\left(t-t_{j}\right)\right\}$ for j=i and a number S−1 of correlative threshold ({j}={i,…,i+S−1}) activated within the subsequent cascade of field increases. Under quasistatic conditions, we define an avalanche as an event starting at some specific threshold activated under external driving such that $\xi \left(t_{i_{0}}\right)=X_{i_{0}}$ and ending on the first instance of the condition:

(5) $$X_{i_{0}+S}>\xi \left(t_{i_{0}+S-1}\right)=\xi \left(t_{i_{0}}\right)+\sum_{i=i_{0}}^{i_{0}+S-1}l_{i},$$

i.e., when all active internal terms $R_{i}$ are fully relaxed. The number of activated thresholds (*S*) marks the size of the avalanche. Given the quasistatic approximation, where $B\left(t_{i_{0}}\right)\approx B\left(t_{i_{0}+\Delta -1}\right)$, Baró and Corral [54] assume a simplified instant response:

(6) $$R_{i}^{u}\left(\tau \right)=l_{i}\;\Theta \left(\tau -\delta \right),$$

where Θ(x) is the step function and the infinitesimal delay time δ structures the avalanche propagation into shells containing all the thresholds activated at the same branching generation. Here, we will refer to this ansatz as the *unimodal mean-field* (UMF) avalanche model, since the relaxation is simplified in a single temporal scale associated with δ.

The resulting avalanches are independent in time with an occurrence rate proportional to ν, and the propagation profile of each avalanche maps onto a memoryless Poisson branching process with branching parameter:

(7) $$\mu_{0}\left(\xi \right)=\nu \left(\xi \right)\times \langle l_{i}\rangle \left(\xi \right),$$

where we use the subindex 0 to indicate that the unimodal mode is a particular case of the bimodal model (Section 2.2) by setting the parameter h=0. The sizes (*S*) of subcritical and critical ($\mu_{0}\leq 1$) avalanches are equivalent to the sizes of subcritical and critical Poisson rooted trees, which are i.i.d. according to a Borel probability mass function (pmf) [56]:

(8) $${\mathrm{pmf}}_{S}\left(S;\mu \right)=\frac{{\left(\mu S\right)}^{S-1}e^{-\mu S}}{S!},$$

with $\mu =\mu_{0}$. For large avalanches and μ→1, Equation (8) can be approximated as ${\mathrm{pmf}}_{S}\left(S\right)\approx S^{-1.5}\mu^{S-1}e^{S(1-\mu )}$, which is consistent with the mean-field universal exponent b=0.5 in Equation (3).

### 2.2. Sub-Avalanches Modeled as Bimodal Relaxation

**Figure 1:**
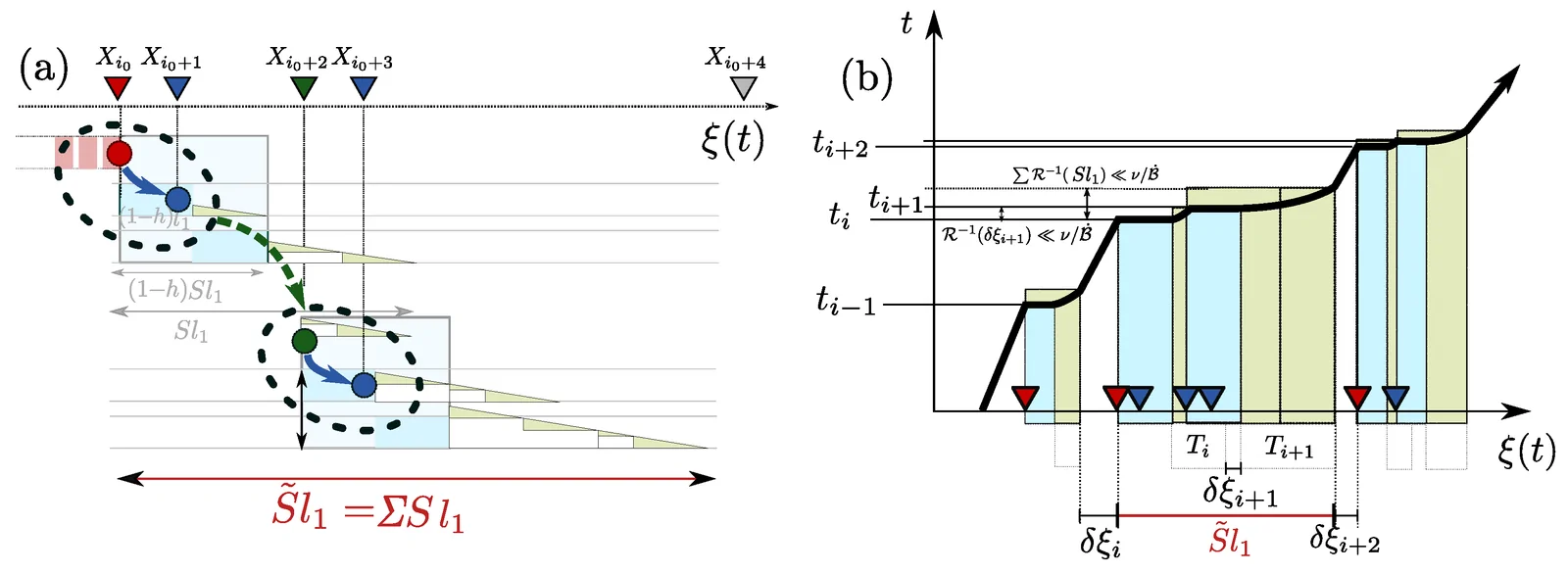
Schematic representation of the aftershock production in the BMF model (**a**) as a branching process in the field span of a single avalanche ($\tilde{S}l_{1}$) and (**b**) as a temporal point process, showing the temporal evolution of ξ(t), in a wider interval containing that avalanche (marked in red). Threshold values are represented in the mean-field (ξ) line as downward pointing triangles. Red triangles are the thresholds initiating avalanches, green are the ones activating sub-avalanches, and blue are activated within a sub-avalanche. Blue and green intervals of ξ represent, respectively, the sudden and slowly relaxed field changes due to two elements in R(τ). The causal links between sub-avalanches are indicated in (**a**) as green dashed arrows.

We associate the existence of event-event triggering with a clear separation of, at least, two characteristic time scales in the internal term $R_{i}\left(\tau \right)$ caused by, at least, two different mechanisms of energy transfer. It is convenient to keep the quasistatic driving approximation; i.e, both mechanisms relax faster than any perceptible change in B(t). In realistic operational scenarios, fast mechanisms are detected as spikes in mean-field change $\dot{\xi }\left(t\right)$ while the low transformation profile caused by slower mechanisms is harder to detect above noise. The resulting evolution of the mean-field ξ(t) within the span of one avalanche, i.e., until all internal terms are fully relaxed (${\dot{R}}_{i}\left(t^{\prime }\right)=0\;\forall i,t^{\prime }>t$), appears split into a series of sudden sub-avalanche events separated by intervals of creep caused by the active slow mechanisms (see Figure 1b). In terms of a point process, we associate such sub-avalanches as the point events that appear clustered and are susceptible to be assigned as aftershocks.

Here, we define the *bimodal mean-field* (BMF) avalanche model as a reduction in the minimal form, considering a simple internal term constituted by the linear superposition of an instantaneous relaxation as in Equation (6) and a monotonous, continuous, and differentiable slow part with a temporal profile G(τ), such that G(0)=0 and G(τ→+∞)=1. (The slow part G(τ) might involve a certain degree of complexity, due to the superposition of different mechanisms with different time scales, without affecting the resulting aftershock statistics, as long as all such scales are well separated from the fast ones. Differences only appear in the temporal statistics of such aftershock production). Since both contributions are normalized between 0 and 1, we represent the internal term in two fractions controlled by 0≤h<1. Thus, the internal term of each threshold can be expressed as

(9) $$R_{i}^{b}\left(\tau \right)=l_{i}\;\left[\;\left(1-h\right)\;\Theta \left(\tau +\delta \right)\;+\;h\;G_{i}\left(\tau \right)\;\right],$$

where δ is an infinitesimal time such that $G_{i}\left(\tau +\delta \right)=G_{i}\left(\tau \right)$ and the slow relaxation is still fast enough such that $G_{i}\left(\tau \right)=1$ while driving remains quasistatic (B(t)=B(t+τ)).

As an example, the BMF model is asymptotically exact in the thermodynamic limit to the avalanche dynamics in a democratic fiber bundle model (DFBM) with generalized Zener elements instead of elastic fibers [45], where the term *h* defines the scale or magnitude of a transient hardening. In particular, the creep compliance of the generalized Zener element can be expressed similarly to Equation (9) as

(10) $$J_{\mathrm{GZ}}\left(\tau \right)=\frac{1}{E}\left\{\left(1-h\right)\Theta \left(\tau +\delta \right)+h\left[1-E_{\alpha }\left(-{\left(\frac{\tau }{\tau_{0}}\right)}^{\alpha }\right)\right]\right\}$$

where $h:=\frac{E_{m}}{E_{m}+E}$, *E* and $E_{m}$ are the elastic moduli defining the primary and secondary elastic elements, and $E_{\alpha }\left(-{\left|x\right|}^{\alpha }\right)$ is the Mittag–Leffler function, resulting from the fractal nature of the springpot element [57] and acting as a power-law relaxation term, which is slower than a memoryless exponential relaxation and recovers the power-law profiles of the temporal kernel in the ETAS model [44,45]. The introduction of transient hardening such as this one in mean-field models is shown to lead to aftershock production both in frictional [55] and fracture [45] models. Here, we focus on the statistical characterization of such aftershock production.

## 3. Stochastic Representation

As in the UMF model [54], we will interpret the dynamics of avalanche and sub-avalanche propagation in the BMF model in terms of stochastic branching processes. We will consider a simplified model with a unique *h*, $l_{i}:=l_{1}$, $G_{i}\left(\tau \right):=G\left(\tau \right)$ and uniform ν, which is a valid approximation at the thermodynamic limit for any infinitesimal subcritical interval [ξ(t),ξ(t)+δξ). Once a threshold $\left\{X_{i}\right\}$ is crossed, the instant field increases as $\xi \left(t_{i}+\delta \right)=\xi \left(t_{i}\right)+\left(1-h\right)l_{1}$. The number of immediately surpassed thresholds due to the fast relaxation is a Poisson number with branching parameter:

(11) $$\mu_{h}:=\left(1-h\right)l_{1}\nu ,$$

analogous to the propagation of avalanches in the UMF in Equation (7) with a correction term (1−h). Once both the slow and fast term relax, the field change due to that specific threshold reaching $\xi \left(t_{0}+\tau \right)-\xi \left(t_{0}\right)=l_{1}$, which recovers the branching parameter $\mu_{0}=l_{1}\nu$ of the UMF model [54]. Consequently, the separation of temporal scales between fast relaxation, slow relaxation and driving results in a structure of nested branching processes in which the avalanche, the size of which is invariant to *h*, is split in sub-avalanche events constituted by the instant cascading activation of thresholds caused by fast (i.e., detectable) relaxation, which are connected by (undetectable) intervals of slow relaxation or creep. The structured dependence between sub-avalanches is similar to the production term ($\Psi_{i}$) assumed in ETAS models.

Figure 1a illustrates the resulting branching process splitting avalanches in sub-avalanches. We will designate as $t_{k}$ the time each sub-avalanche event (*k*) starts. Consider that a first threshold $X_{i_{0}}$ is activated due to the slow driving at time $t_{i}$, when $\xi \left(t_{i}\right)=X_{i_{0}}$, initiating both the root sub-avalanche (event *i* in the point sequence) and the triggering tree or full avalanche. At time $t_{i}+\delta$, i.e., after the fast relaxation, a number $K_{1}$ of new thresholds fall behind the mean-field value:

(12) $$\xi \left(t_{i}+\delta \right)=\xi \left(t_{i}\right)+\left(1-h\right)l_{1},$$

and are activated due to the fast relaxation. In Figure 1, $K_{1}=1$ and only $\left\{X_{i_{0}+1}\right\}$ is activated. The process is recursive, leading to the internal propagation within the sub-avalanche. At time $t_{i}+g\delta$, a new shell (or generation) *g* constituted by a number $K_{g}$ of thresholds is activated due to the increase:

(13) $$\xi \left(t_{i}+g\delta \right)=\xi \left[t_{i}+\left(g-1\right)\delta \right]+\left(1-h\right)l_{1}K_{g-1}.$$

Therefore, the propagation of the first sub-avalanche is, as expected, a Poisson branching process that is equivalent to the propagation of avalanches in the unimodal model, leading to Borel distributed sub-avalanche sizes ($S_{i}$), as in Equation (8) but now with $\mu =\mu_{h}$. Following the avalanche propagation, after all the fast internal terms are relaxed, the next threshold might, or might not, be reached before all previous slow internal terms are fully relaxed (e.g., the case of $\left\{X_{i_{0}+2}\right\}$ in Figure 1). If any, the first aftershock corresponds to the next sub-avalanche event i+1 initiated at time $t_{i+1}$ at which the next threshold $X_{{\left(i+1\right)}_{0}}$ is reached by slow relaxation. For example, the activation of $X_{{\left(i+1\right)}_{0}}=X_{i_{0}+2}$ in Figure 1 is shown in Equation (14). (The equation can be inverted to determine $t_{i+1}$, thereby resolving the occurrence times of consecutive sub-avalanche events. This relation becomes more complex, although it remains numerically solvable, when considering the concurrent relaxation of several sub-avalanche events [45]).

(14) $$\xi \left(t_{i+1}\right)=\xi \left(t_{i}\right)+S_{i}\left(1-h\right)l_{1}+S_{i}hl_{1}G\left(t_{i+1}-t_{i}\right)=X_{{\left(i+1\right)}_{0}}.$$

Right after the activation of $X_{{\left(i+1\right)}_{0}}$, the field increases as $\xi \left(t_{i+1}\right)=\xi \left(t_{i+1}+\delta \right)+\left(1-h\right)l_{1}$, leading to the same branching process for the root in Equation (13) with the same branching ratio in Equation (11). Therefore, sub-avalanche sizes are i.i.d. as Equation (8) with $\mu =\mu_{h}$.

## 4. Aftershock Production

The main difference between the UMF and the BMF is the introduction, in the latter, of clustering in the time series of sub-avalanche events. As expected, the final field value at the end of the avalanche after all responses, fast and slow, are relaxed reads $\xi \left(t\to \infty \right)=\xi \left(t_{i,0}\right)\;+\;l_{1}\sum_{k}S_{k},$ where *k* contain now all sub-avalanche events, and leading to a state equivalent to consider the UMF. Therefore, the size ($\tilde{S}$) of the avalanche is invariant to *h*, and the effect of *h* is the splitting of the avalanche in a sequence of inter-event creep intervals and instant individual sub-avalanche events of size $S_{k}$, such that $\tilde{S}=\sum_{k}S_{k}$. The resulting point process can be interpreted both in terms of the mean-field (ξ) and time (*t*) units (x and y axes in Figure 1b). Due to the independence of $\left\{X_{i}\right\}$, the field difference separating the end and initiation of consecutive sub-avalanche events, both background or aftershock, are exponentially distributed in units of ξ with frequency ν. Therefore, temporal clustering appears in the form of aftershock production only in the temporal point-process representation, not in the mean-field line where $\left\{X_{i}\right\}$ are i.i.d. Whilst avalanches themselves are temporally independent, only conditioned by the driving rate $\dot{B}\left(t\right)$, sub-avalanche events within the same avalanche are temporally correlated given the functional form of G(τ). Such temporal correlations, leading to a process similar to self-excitation, might be interpreted as the result of univocal causal links connecting each parent event destabilizing a field interval and the offspring initiated by $\left\{X_{i}\right\}$ values within that interval. This is considering the span between the relaxed state of all previously activated thresholds in the simulation right before and right after the parent sub-avalanche. Following this stability criteria, the first sub-avalanche event within an avalanche corresponds to a background event, in terms of ETAS models, or a root, in terms of branching processes, which is activated by the crossing of threshold $X_{i}$ due to B(t) if all internal terms are quiescent or fully relaxed. A sub-avalanche *j* is defined to be triggered by a previous sub-avalanche *i* of size $S_{i}$ starting at a time $t_{i}$ if and only if the threshold value $X_{j}$ initiating the sub-avalanche *j* fits within an interval:

(15) $$B\left(t_{i}\right)+\sum_{k<i}S_{k}l_{1}<X_{j}\leq B\left(t_{i}\right)+\sum_{k<i}S_{k}l_{1}+S_{i}l_{1},$$

meaning that the field increase caused by $S_{i}$ is the first one sufficient to push the field ξ across the value of $X_{j}$ at some finite time before the driving resumes. As illustrated in Figure 1a, a background sub-avalanche 0 of size $S_{0}$ can trigger a number $N_{1}$ of first-generation (d=1) aftershocks. A generation *d*, consisting of $N_{d}$ of sub-avalanche events, will trigger in its turn a number $N_{d+1}:=\sum_{j}^{N_{d}}N_{j}$ sub-avalanche events constituting generation d+1, accounting for the $N_{j}$ direct aftershocks of each sub-avalanche event *j* within $N_{d}$.

### 4.1. Aftershock Production in Terms of Field Interval

**Figure 2:**
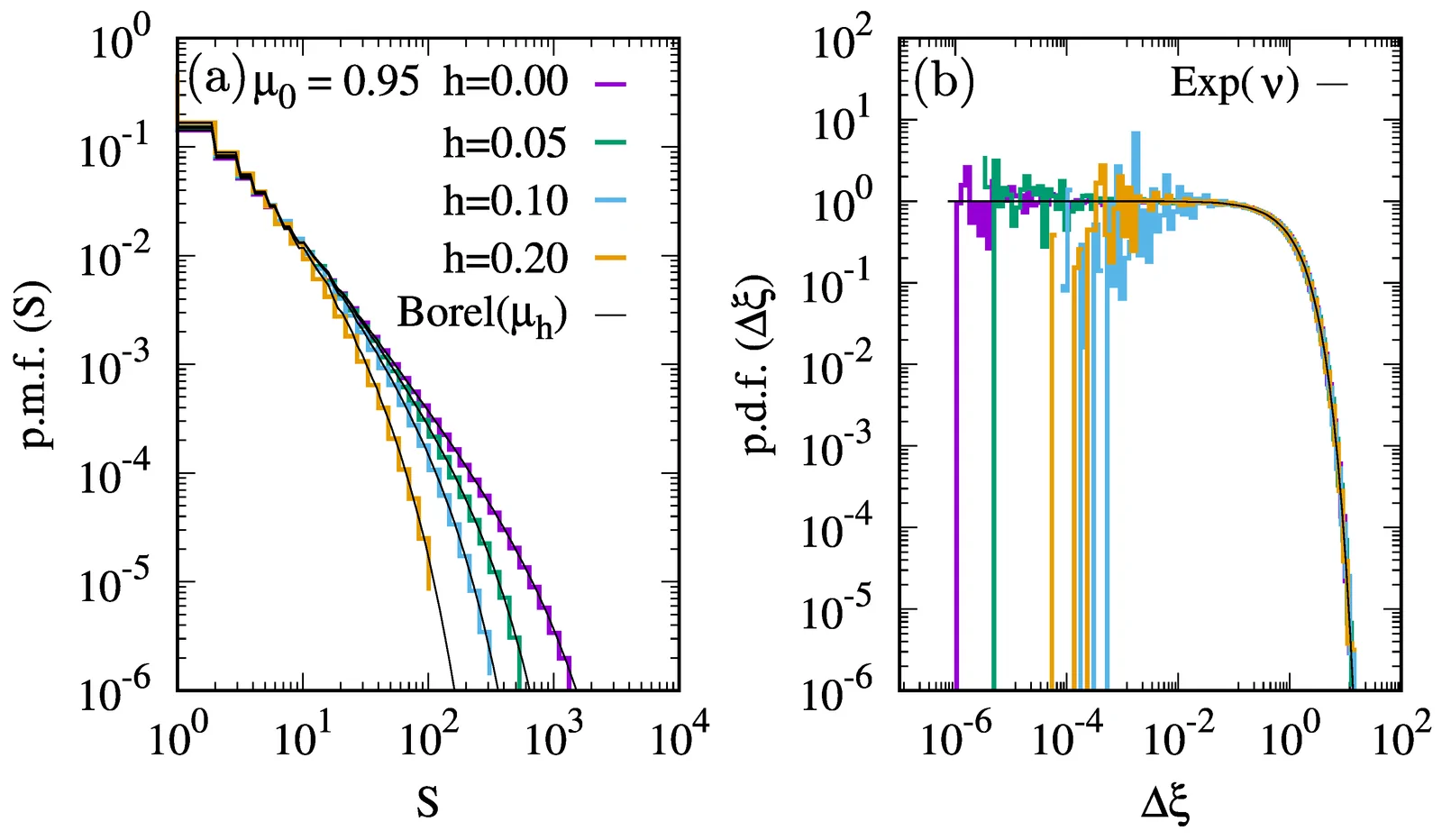
Sub-avalanche distributions extracted from raw BMF simulations represented in logarithmic bins: (**a**) distribution of sub-avalanche sizes (*S*) compared to the predicted Borel dsitributions in Equation (8); (**b**) distribution of field intervals between sequential sub-avalanches and the predicted Poisson process with a rate coinciding with the imposed parameter ν=1.

**Figure 3:**
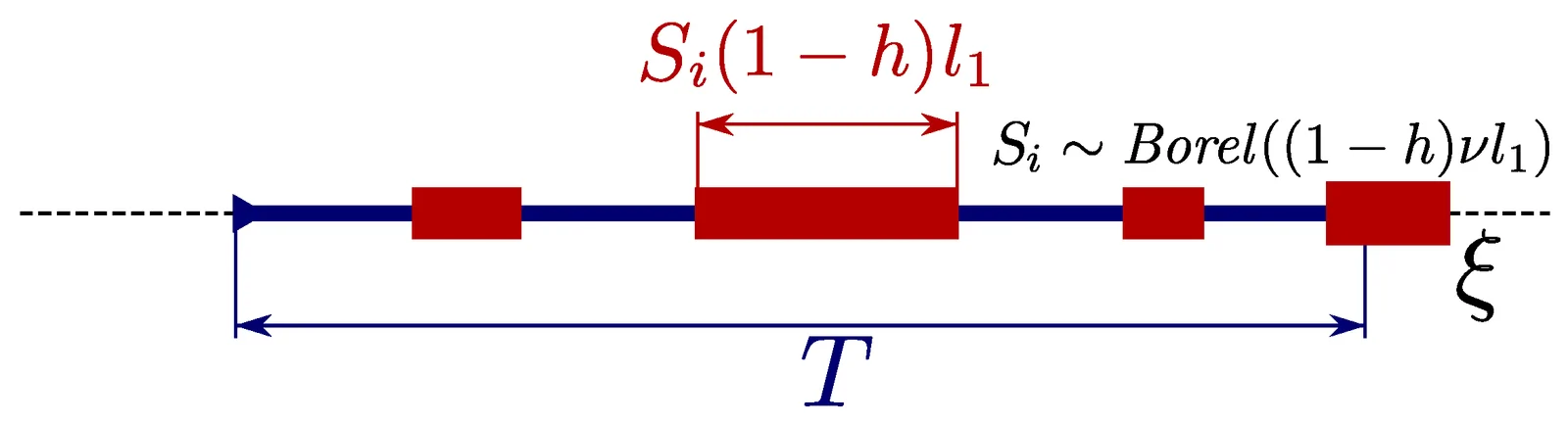
Schematic representation of the renewal process describing the statistics of direct aftershocks. Within a mean-field interval *T* (blue line), we can fit a number *N* of {i} independent events with frequency ν and refractory field-intervals $\left\{S_{i}\left(1-h\right)l_{1}\right\}$ (wide red lines) being $S_{i}$ Borel distributed, as in Equation (8) with parameter $\mu_{h}:=\left(1-h\right)\nu l_{1}$.

We define $T_{j}$ as the effective field interval within which aftershocks of sub-avalanche *j* can be produced. As shown in Section 4.2, the general relation between $T_{j}$ and Equation (8) is not a trivial matter. The number of direct aftershocks triggered by *j* is the number of sub-avalanches that are initiated within this interval $T_{j}$. Since we consider the same parameters $l_{1}$, ν and *h*, all sub-avalanche events are i.i.d. with sizes ($S_{i}$) distributed as in Equation (8) with the branching ratio in Equation (11), as shown in Figure 2a. Hence, each of these aftershocks causes a sudden increase in the field $S_{i}\left(1-h\right)l_{1}$ within which no new sub-avalanches can be initiated, since all $\left\{X_{k}\right\}$ activated within belong to event *i*. Therefore, the sequence of direct aftershocks can statistically be modeled as a renewal process of independent events with stochastic refractory mean-field intervals, or *times* in the traditional language, as schematically illustrated in Figure 3. The pmf of $N_{j}$ is a Poisson number excluding the refractory mean-field intervals.

We can derive an approximate solution to the statistics of N|T for large numbers. As verified with the statistical distributions shown in Figure 2, the mean-field intervals between consecutive sub-avalanches (Δξ) account for the i.i.d. inter-event intervals ν and refractory times:

(16) $$\Delta \xi ∼\mathrm{Exp}\left(\nu \right)+\left(1-h\right)l_{1}\mathrm{Borel}\left(\mu_{h}\right),$$

where Exp(ν) and $\mathrm{Borel}(\mu_{h})$ are i.i.d. numbers from the independent exponential distribution for random events and the Borel distribution for sub-avalanche sizes, respectively. Due to the statistical independence between the two terms, the cumulants are additive. Thus,

(17) $$\begin{array}{c} E\left(\Delta \xi \right)=\frac{1}{\nu }\left(1+\frac{\mu_{h}}{1-\mu_{h}}\right)=\frac{1}{\nu }\frac{1}{\left(1-\mu_{h}\right)}, \end{array}$$

(18) $$\begin{array}{c} \mathrm{Var}\left(\Delta \xi \right)=\frac{1}{\nu^{2}}\left[1+{\left(\frac{\mu_{h}}{1-\mu_{h}}\right)}^{3}\right]. \end{array}$$

The central limit theorem (CLT) provides a solution to renewal counting processes for large numbers [58]. Considering Equations (17) and (18), the approximate number of aftershocks produced in a field interval *T* follows a symmetric Gaussian distribution with parameters mean $\mu_{N}$ and variance $\sigma_{N}^{2}$ such that

(19) $$\begin{array}{c} n_{T}\left(\nu T;\mu_{h}\right):=\mu_{N}\left(T;\mu_{h}\right)\approx \frac{T}{E(\Delta \xi )}=\left(1-\mu_{h}\right)\nu T, \end{array}$$

(20) $$\begin{array}{c} \sigma_{N}^{2}\left(T;\mu_{h}\right)\approx \frac{\mathrm{Var}(\Delta \xi )}{{\left[E\left(\Delta \xi \right)\right]}^{3}}=\left[\frac{1}{4}+3{\left(\mu_{h}-\frac{1}{2}\right)}^{2}\right]\nu T. \end{array}$$

**Figure 4:**
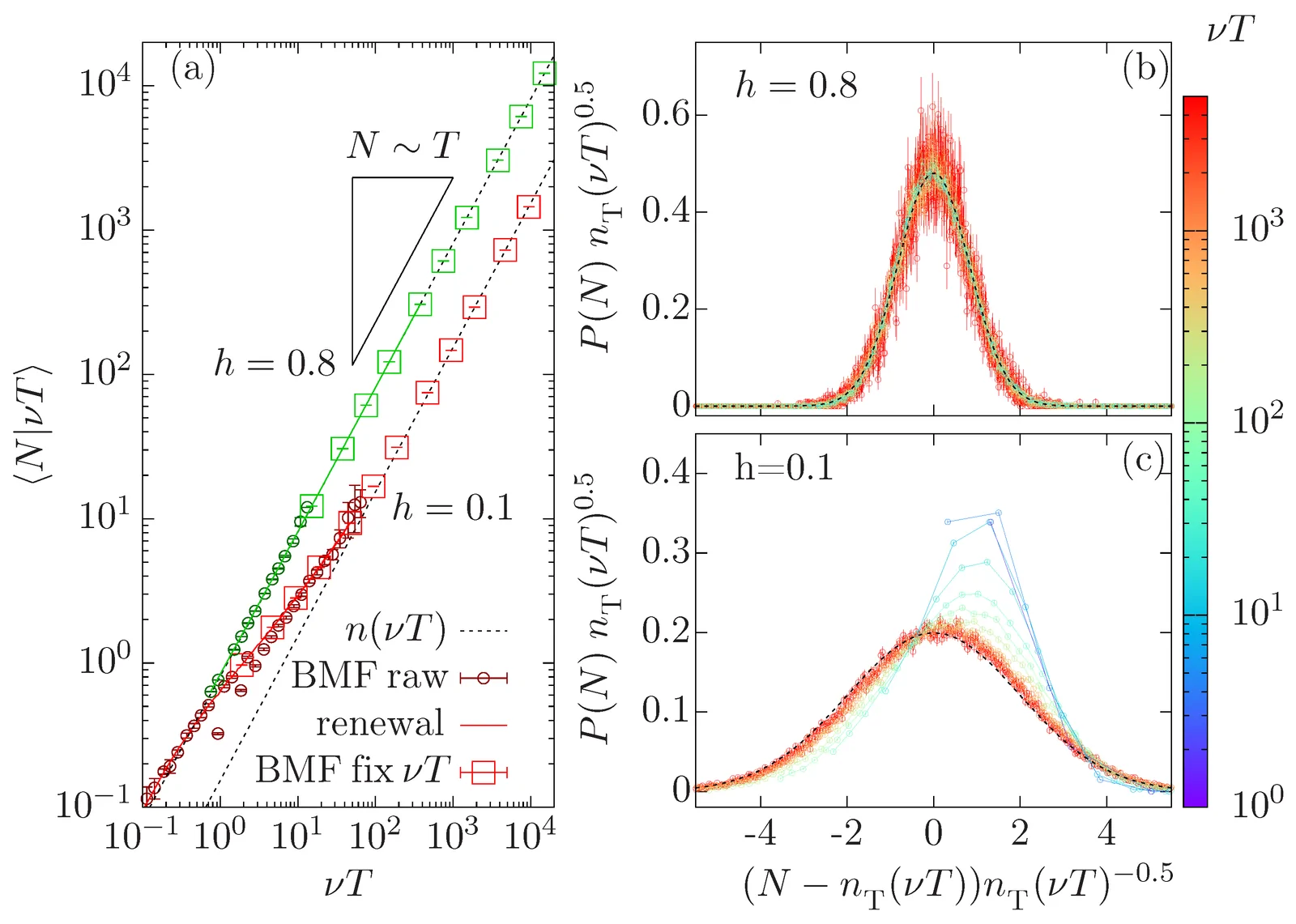
Conditional averages 〈N|νT〉 of direct aftershock production for $l_{1}\nu =0.940$ and h=0.8 (green) and h=0.1 (red). (**a**) Small circles show the averages obtained by raw BMF simulations sampling $10^{7}$ random thresholds; large boxes show simulations of $10^{5}$ independent sequences of events within an imposed νT; solid lines show the averages of $10^{5}$ simulations of the renewal model for each value of νT; dashed lines show the CLT limit $n_{T}\left(\nu T\right)$ from Equation (19). Scaling collapses of P(N|νT) are shown (**b**) for h=0.8 and (**c**) for h=0.1. Black dashed lines show the Gaussian CLT solution from Equation (21) imposing Equation (20).

Figure 4a compares the conditional averages of direct aftershocks 〈N|νT〉 obtained in raw BMF simulations, in small dots, the renewal model from Equation (16), in solid colored lines, and the asymptotic CLT limit $n_{T}\left(\nu T\right)$ in Equation (19), in dashed black lines. We show the results of two different *h* values (h=0.1 and 0.8) at a high avalanche branching ratio ($\mu_{0}=\nu l_{1}=0.94$). For the raw simulation, we only consider background events, for which the interval has a linear dependence with sizes $T_{j}=hl_{1}S_{j}$ (see Section 4.2). Since sub-avalanche sizes in the BMF are subcritical, the span of *T* values reachable in raw simulations is limited to a characteristic upper cutoff derived from the Borel distribution in Equation (8). We verify the convergence to the asymptotic approximation for long *T* and provide predictions for rare extreme events by directly simulating sub-avalanche sequences imposing fixed *T* values (large squares). The BMF simulations by the two methods are indistinguishable from the renewal model in Equation (16) for all sizes. The CLT limit $n_{T}\left(\nu T\right)$ stands even for small *T* values, except in regimes where large sub-avalanches are statistically significant, i.e., close to the critical branching considering both $l_{1}\nu ⪅1$ and h⪆0 (e.g., red in Figure 4a). In Figure 4b,c, we verify the agreement for the different *h* values with a scaling relation:

(21) $$P\left(N|\nu T\right)=\frac{1}{\sqrt{n_{T}\left(\nu T\right)}}\;\Phi \left[\frac{N-n_{T}\left(\nu T_{j}\right)}{\sqrt{n_{T}\left(\nu T_{j}\right)}}\right].$$

The scaling function Φ(x) agrees with the Gaussian approximation quite beyond the CLT limit except where aftershock production is low, e.g., for small parents at high branching ratio ($l_{1}\nu =0.94$ and h=0.1 in Figure 4c). The width of the distribution scales on $\mu_{h}$ as predicted for the variance in Equation (20).

**Figure 5:**
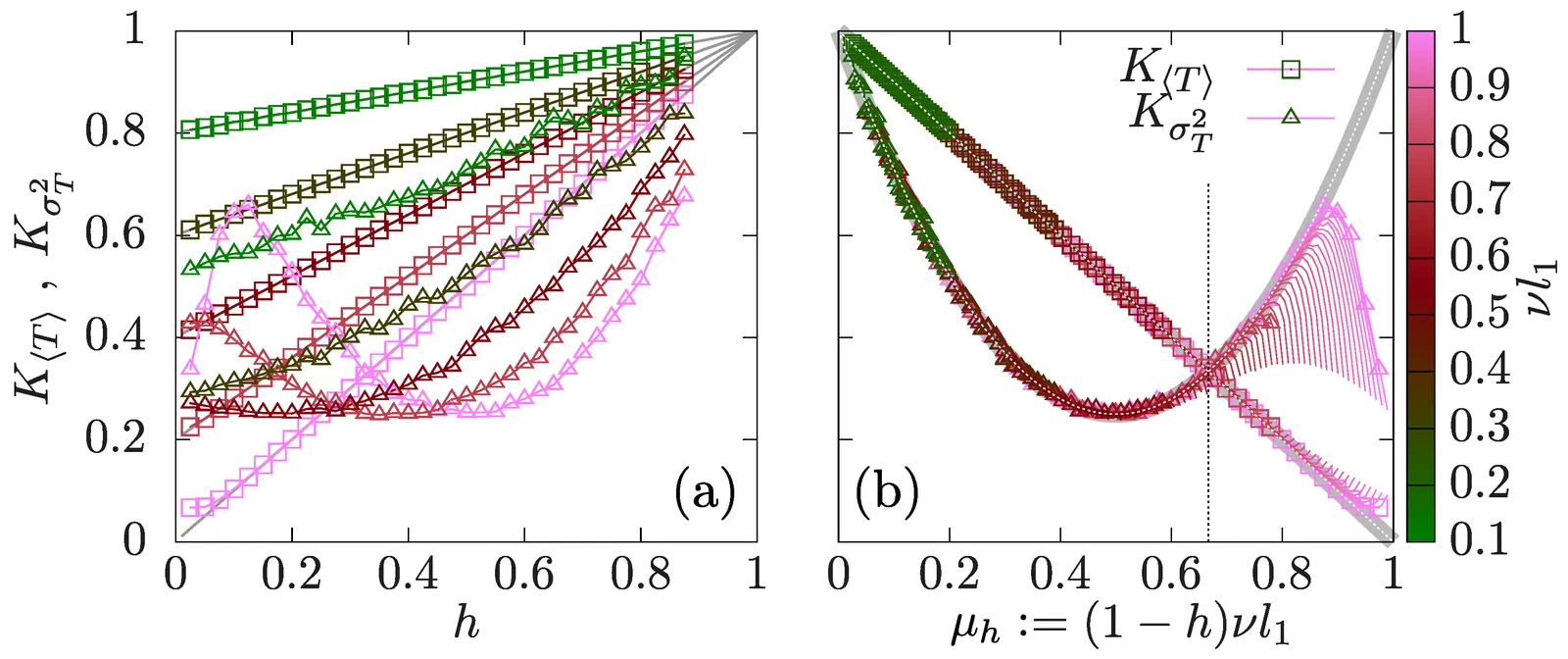
Representation of *K* for mean and variance as function of *h* and $\nu l_{1}$. Values *K* estimated from the linear relation n(νT)=KνT are shown in squares ($K_{\langle T\rangle }$) and triangles ($K_{\sigma_{T}^{2}}$) with color-scheme matching the $\nu l_{1}$ values: (**a**) as function of *h*; (**b**) as function of $\mu_{h}$. Gray lines show the expected results from Equations (22) in both (**a**) and (**b**), and (23) in (**b**).

The universal dependence of aftershock production in terms of $\mu_{h}$ is further exposed in Figure 5. The expected value from Equation (19) can be expressed in terms of the productivity law in Equation (2) as linear asymptotic dependence $n_{T}\left(\nu T\right)=K_{\langle T\rangle }\left(\mu_{h}\right)\times \nu T$ with

(22) $$K_{\langle T\rangle }\left(\mu_{h}\right)\approx 1-\mu_{h}.$$

In a similar way, we can express the variance in Equation (20) in terms of the linear dependence $\sigma_{N}^{2}\left(T\right)=K_{\sigma_{N}^{2}}\left(\mu_{h}\right)\times \nu T$, also depending only on the branching parameter ($\mu_{h}$) with the parabolic shape:

(23) $$K_{\sigma_{T}^{2}}\left(\mu_{h}\right)\approx 0.25+3{\left(\mu_{h}-0.5\right)}^{2}.$$

Figure 5a shows the estimates of $K_{\langle T\rangle }\left(h,\mu_{0}\right)$ by fitting the cumulants from independent aftershock sequences at different fixed *T* (e.g., the large boxes in Figure 4a) up to $T=10^{4}$ as a linear regression by linear least squares (LLS). Each line corresponds to the dependence on *h* given different values of $\mu_{0}=\nu l_{1}$. Figure 5b shows the collapse of both $n_{T}$ and $\sigma_{T}^{2}$ as a function of $\mu_{h}$. The collapse agrees with the large-number approximation in Equations (22) and (23) except for large branching parameters $\mu_{0}$ and low transient hardening h≲0.1 for which aftershocks are scarce. Only for $\mu_{h}=2/3$ is the variance of aftershock production compatible with the null Poisson hypothesis ($K_{\langle T\rangle }=K_{\sigma_{T}^{2}}$) considered in the point process interpretation of ETAS models. That point marks a transition between under- and overdispersion. The Fano factor $F\left(\mu_{h}\right)=\sigma_{T}^{2}\left(\mu_{h}\right)/n_{T}\left(\mu_{h}\right)$ decreases as function of $\mu_{h}$ down to a minimum value $F_{\min}=2\sqrt{3}-3\approx 0.46$ for $\mu_{h}=1-1/\sqrt{3}\approx 0.42$ before a progressive increase, crossing the transition point, toward a divergence at $\mu_{h}\to 1$.

### 4.2. Aftershock Production in Terms of Sub-Avalanche Size

**Figure 6:**
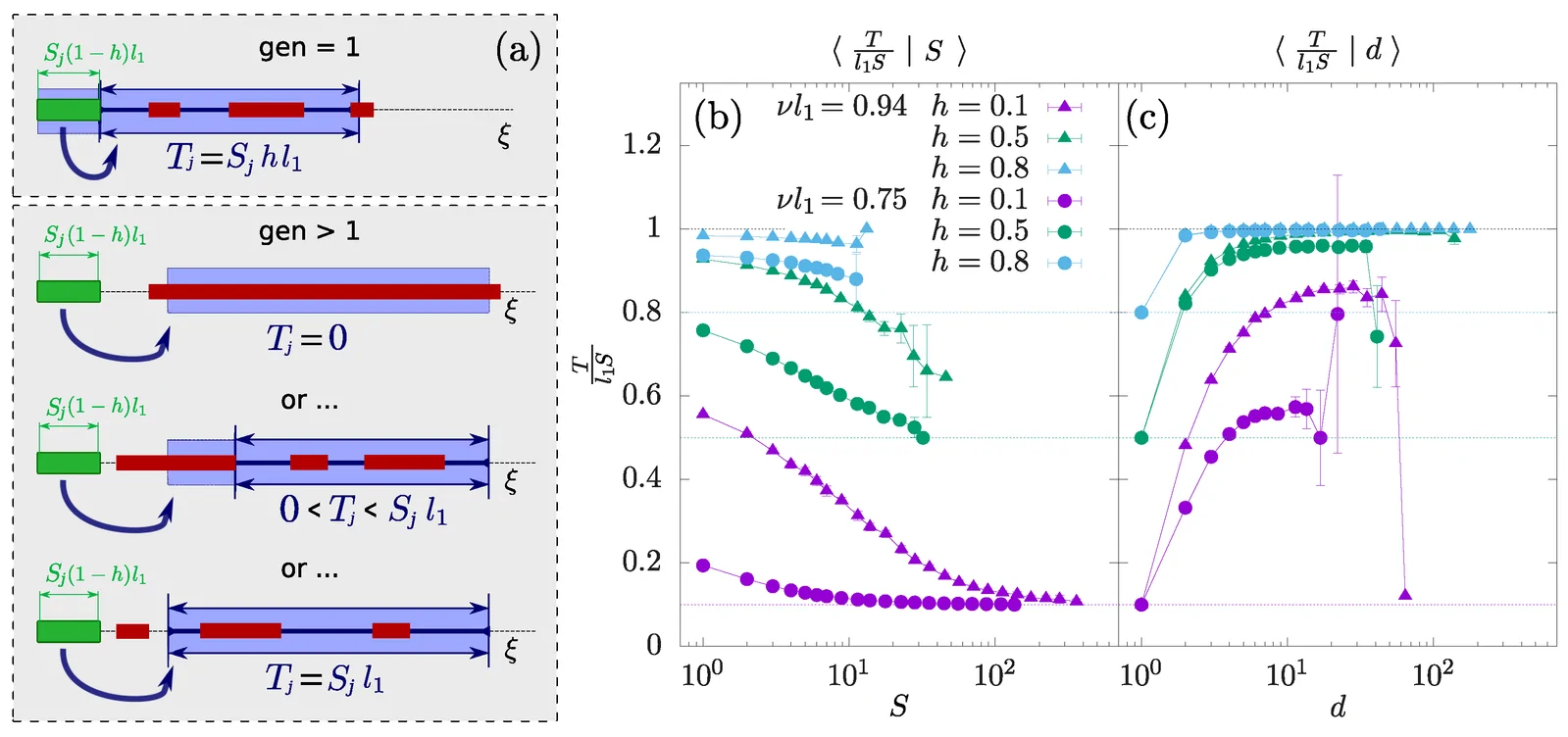
(**a**) Possible screening scenarios for the effective $T_{j}$ given a parent event sized $S_{j}$ and depending on the aftershock generation. The shaded blue area corresponds always to $S_{j}l_{1}$. First-generation aftershocks always occur within an interval $T_{j}=hl_{i}S_{j}$. For higher generations, the interval $T_{j}$ is stochastic and constrained between 0 and $S_{j}l_{1}$ depending on whether the situation was total, partial or no screening due to previous events. (**b**) Dependence of the mean length of unscreened field intervals for aftershock production (*T*) on the generational depths of such aftershocks (*d*) and (**c**) the size of the parent (*S*). The conditional averages are normalized by the upper bound $l_{1}S$. The values 1−h expected for background events following Equation (24) are marked with colored dash-lines. Black dashed lines correspond to the limit $T_{j}=l_{1}S_{j}$.

The solutions in terms of $T_{j}$ derive from the renewal model in Equation (16), which imposes a Poisson field increase between the end of a sub-avalanche and the onset of the next. This implies that the onset of the first aftershock is Poisson. This condition requires that $T_{j}$ excludes the initial offsets caused by the refractory intervals $\left(1-h\right)l_{1}S_{k}$ of the same event *j* or any other sub-avalanche event, the aftershock of an event previous to *j*, screening the interval before any aftershock of *j* can occur. For example, we consider the intervals $T_{i}$ and $T_{i+1}$ in Figure 1a. If *j* is the root of an aftershock tree, the interval in Equation (15) starts at the onset of the parent event. Therefore,

(24) $$T_{j}^{\left(g=1\right)}=l_{1}S_{j}-\left(1-h\right)l_{1}S_{j}=hl_{1}S_{j},$$

i.e., the interval slowly relaxed by that event. This implies that the expression for *T* in Equation (19) directly provides a closed mathematical solution for first-generation aftershocks (d=1) considering Equation (24). On the contrary, the interval in Equation (15) for higher generation aftershocks (d>1) is detached from the onset of the parent. Such an interval might be screened, or not, by itself or other events within the same aftershock tree, causing a non-trivial generational memory in the aftershock branching process. The different possible scenarios are schematically represented in Figure 6. In general, $T_{j}$ is bounded within:

(25) $$0\leq T_{j}\leq l_{1}S_{j}.$$

**Figure 7:**
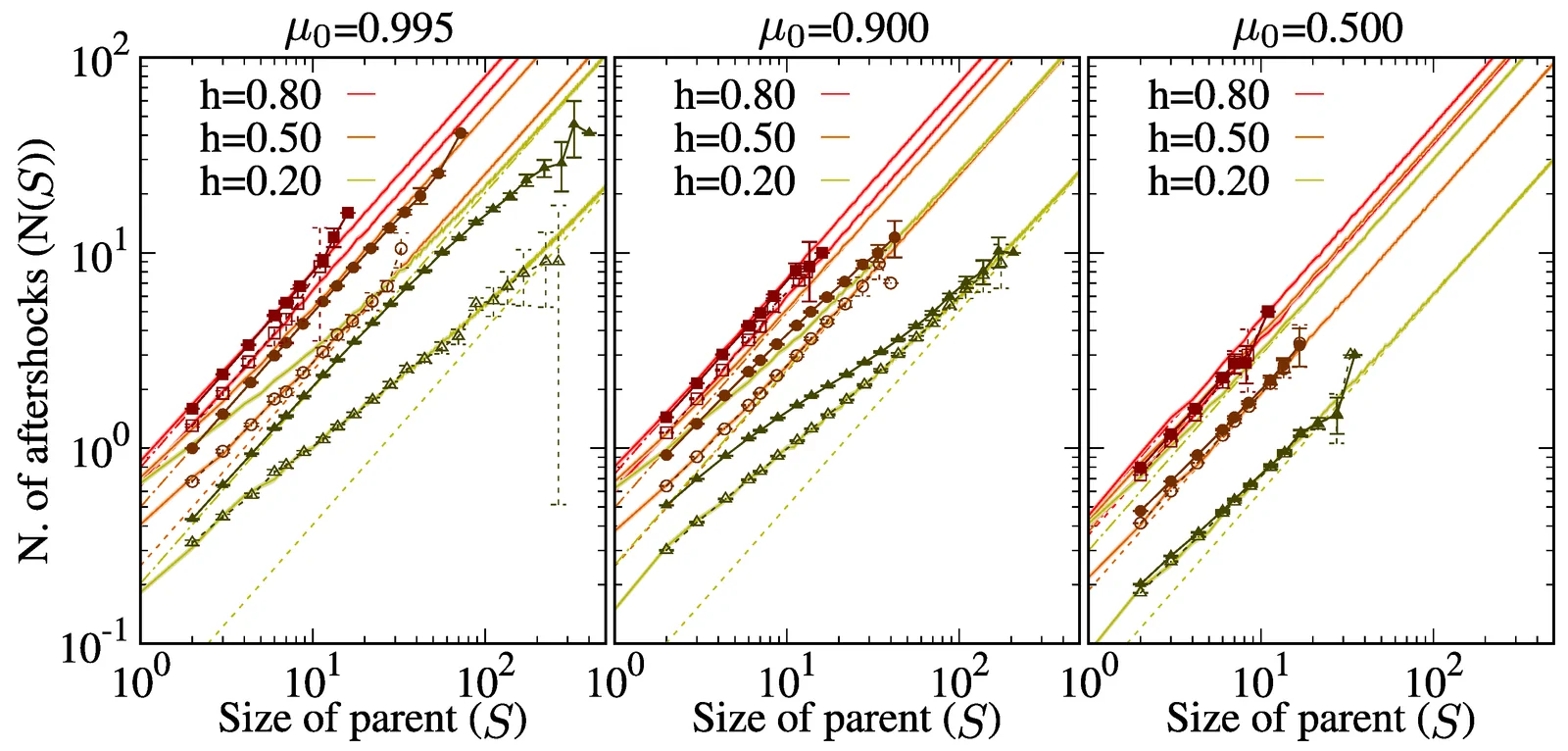
Effective productivity law of direct aftershocks in terms of parent size for $\mu_{0}=0.9$ and h=0.2 from conditional averages $\langle N_{j}|S_{j}\rangle$ for d=1 (empty symbols) and for all *d* values (filled symbols). Solid lines show the expected values for the renewal model in Equation (16) for first-generation aftershocks ($T=\left(1-h\right)l_{1}S$) and the upper limit ($T=l_{1}S$). Dashed lines show the asymptotic CLT solution in Equation (19) considering both limits.

The right panels in Figure 6 show the dependencies of average *T* normalized by the size of events ($l_{1}S$). The memory effects tend to level off at high generations toward an unspecified value higher than the one for d=1 (Figure 6c). On the contrary, for large *S*, i.e., large *T*, the relation approaches the one for d=1 (Figure 6b). The aftershock production over all generations is compared to first-generation aftershocks in Figure 7 for a reduced selection of $\mu_{0}$ and *h* values. Four theoretical lines are shown for each data-set. Lower solid lines corresponds to the numerical solution of Equation (16) assuming Equation (24), which matches the theoretical prediction for first-generation aftershocks. Upper solid lines represent the theoretical limit $T_{j}=l_{1}S_{j}$. Dashed lines mark the CLT asymptotic behavior for both cases. Apart from the perfect match between first-generation aftershocks and the renewal model assuming Equation (24), the simulation results considering all generations are encased between the two renewal models (solid lines). This is consistent with Figure 6c, where the effective interval *T* is enclosed between $hl_{1}S$ and $l_{1}S$, which is a gap that narrows as *h* approaches one. Therefore, the asymptotic CLT limit forcedly leads to N∝S even considering all generations. We also observe that although the global production always approaches the first-generation solution, the approach to the CLT limit is slower as the model approaches criticality ($\mu_{0}\to 1$) where Borel refractory times become broad.

**Figure 8:**
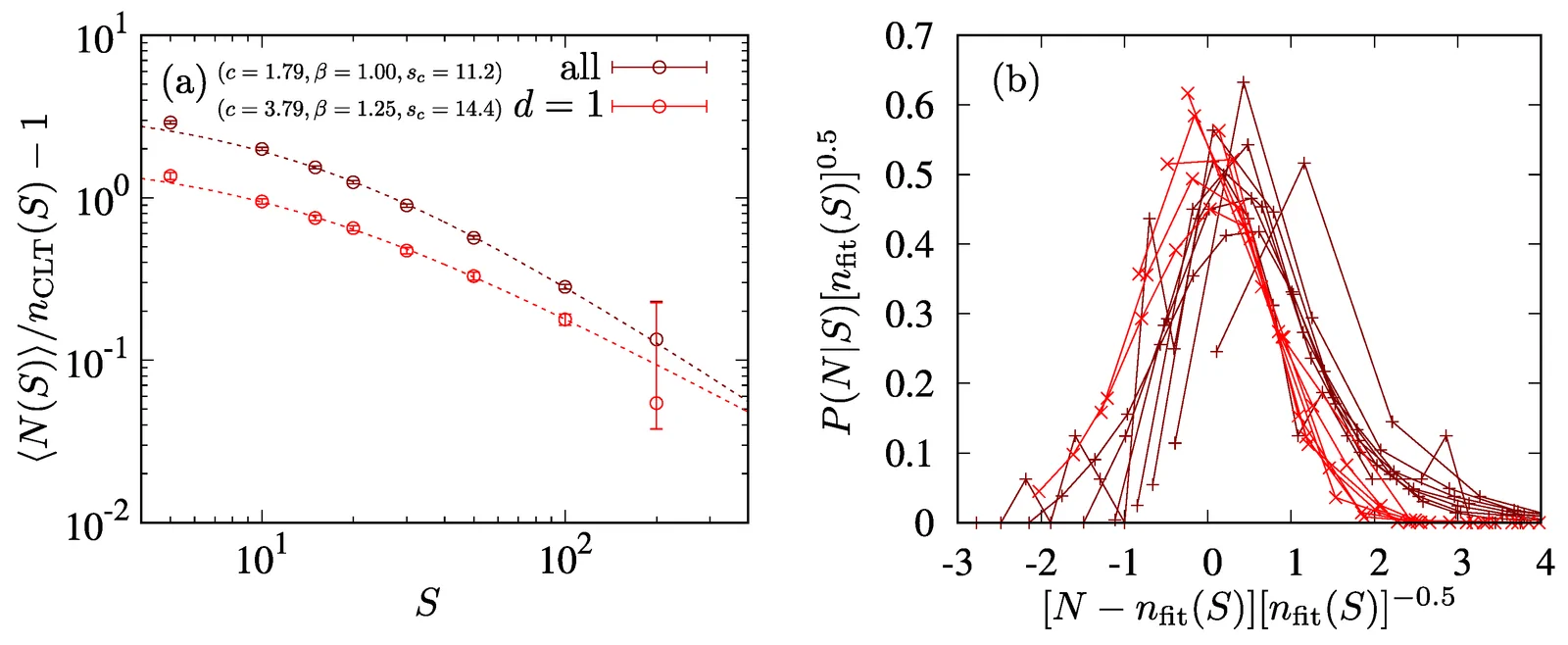
Simulation results of BMF for $\mu_{0}=0.900$, h=0.2 and $N=10^{7}$ thresholds compared with the ansatz in Equation (26) for first-generation aftershocks (d=1) and all generations. (**a**) The approach of $\langle N_{j}|\Delta_{j}\rangle$ toward $n_{\mathrm{CLT}}$ represented in the scaled difference between them. Dashed lines show the best fit for $n_{\mathrm{fit}}\left(S\right)$. (**b**) Scaling collapse of distributions P(N|S), as in Figure 4c, considering the scaling parameter from Equation (26).

The higher differences with first-generation aftershocks are observed in the situation with higher branching, approaching the scale-free limit $\mu_{h}\to 1$. Around this limit, an estimation of the productivity laws within a narrow interval of *S* could render effective α exponents below 1. We explore the possible range of such an effective exponent when estimated by LLS in a log-log scale. (In order to address statistical artifacts below the CLT limit, a more precise maximum-likelihood estimation of parameters α and *K* is advised [33], but it should account for the non-Poissonian nature of the underlying renewal counting process, which is beyond the scope of this paper.) In Figure 8a, we fit the following heuristic correction to the average numbers for $\mu_{0}=0.9$, h=0.2, considering the renewal-counting-process CLT ($n_{\mathrm{CLT}}$) in Equation (22) with $T=hl_{1}S$:

(26) $$\langle N|S\rangle \approx n_{\mathrm{fit}}\left(S\right):=\left(1+c{\left(1+S/s_{c}\right)}^{-\beta }\right)n_{\mathrm{CLT}}\left(S\right),$$

where *c*, $s_{c}$ and β are positively defined free parameters. The Gaussian scaling for the CLT limit in Equation (21) around this ansatz (26) is significant and accurate, considering the reduced statistics, even for small numbers (Figure 8b). We can approximate an effective productivity law given by an LLS fitting in the log-log space as

(27) $$\alpha_{\mathrm{eff}}\left(S\right):=\frac{\partial \ln(N)}{\partial \ln(S)}=1-\frac{\beta c\;S/s_{c}}{\left(1+S/s_{c}\right)\left[{\left(1+S/s_{c}\right)}^{\beta }+c\right]}.$$

This expression has a single minimum at $S_{m}$ given $c{\left(1-S_{m}/s_{c}\right)}^{-\beta }=b\left(1-S_{m}/s_{c}\right)-b+1$. The example in Figure 8, close to the large branching limit, accepts a minimum $\alpha_{\mathrm{eff}}\left(S_{m}\right)\approx 0.75$ for first-generation aftershocks and $\alpha_{\mathrm{eff}}\left(S_{m}\right)\approx 0.60$ for all aftershocks.

## 5. Discussion

We obtained a closed asymptotic solution for aftershock production in the mean-field model owing to the direct mapping between avalanches and branching processes, which allows the interpretation of aftershock production as the renewal process in Equation (16), that would be otherwise difficult to obtain in more realistic low-dimensional systems. This mean-field approximation has its own limitations to explain field observations.

Since avalanche growth is constrained by the branching condition $\mu_{0}\leq 1$, the sizes of sub-avalanches, with branching parameter $\mu_{h}=\left(1-h\right)\mu_{0}$, are subcritical. Therefore, it is mathematically impossible within the present formulation to reproduce both aftershock production and the scale-free Gutenberg–Richter law from Equation (3). Another important limitation of the mean-field approximation is the kind of aftershock-tree structures that can be obtained. By construction, all aftershocks in the BMF model are temporally ordered. Aftershocks of event *i* will occur after all aftershocks of event j<i and before all aftershocks of event j>i. The resulting “combed” tree structures are unlikely to occur in real catalogs, where local field changes will activate independent branches involving different regions. Attempts to compare branching topologies with field observations [28] are therefore forfeit.

Finally, we highlight that causal connections between sub-avalanche events are established through stability criteria within the micromechanics rather than through the spatiotemporal distance or other metrics commonly used in earthquake catalogs [59,60]. The distinct definitions of causality may partly account for discrepancies in the inferred aftershock statistics. Since spatial information is absent in the mean-field formulation, aftershock identification based on temporal proximity alone would produce a sequential genealogy, where each event is attributed, if any, to the immediately preceding one. This alone is sufficient to render useless any comparative attempt between the two identification approaches. Tectonophysical models, such as the clock advance model [32] or the solid seismicity postulate [33], assume similar stability criteria. There, the spatial density of direct aftershocks is assumed to be proportional to the Coulumb stress change. We interpret the metric criteria used in field catalogs as an operational inference of the underlying causal network based on more fundamental physics rather than as unique definitions of causality.

Mean-field models commonly share a universality class with characteristic scale-free exponents of avalanche statistics, e.g., b=1/2, which is a consequence of the lack of memory in avalanche profiles [54]. The range of scale-free exponents is known to broaden when considering low-dimensional systems and it is generally non-universal, exhibiting parameter dependencies on aspects such as dissipation, interaction ranges and structures. This is the case of earthquakes where mechanistic [61,62], regional [63,64], or temporal [55,65,66,67] dependencies on b-value are often discussed, which are always around ≈0.7, higher than mean-field models, when measured in earthquake moment magnitudes or displacement [68]. We highlight that within the idealized pure scale-free scenario, aftershock production is determined by the ratio of α/b and a *K* production term [7,27,28]. Therefore, α and b-values are not independently fundamental to the statistics of aftershock production. However, we cannot discard such non-universality in low-dimensional systems as an alternative mechanism tuning *b* or α toward the self-similar aftershock production. Acknowledging the limitations of the mean-field approximation, the present results represent a counterexample indicating that stress-relaxation dynamics alone do not generically enforce the self-similar condition imposed in the BASS limit (α=b). We have shown how lower effective α values are possible below the CLT limit both when considering the complete aftershock sequence and when restricting the analysis to first-generation aftershocks. Close to the self-similar limit ($\mu_{0}=1$ and h→0), the convergence toward the asymptotic is slower. Therefore, the observation of a transient exponent α<1 is plausible, even for relatively strong parent events, but it is still significantly higher than the BASS limit α=0.5. Similarly, Mignan [33] reports a higher exponent α≈1, which is self-similar in terms of *m*, for southern California when restricting the study to large parents (m>6), in contrast to the lower α-values where statistics are affected by low aftershock production numbers (2<m<6). This behavior is opposed to the theoretical one, where the geometric constrains toward a 2D plane appear, precisely for strong events, finding a higher ratio (α/b=1.5) for weak earthquakes in an unrestricted 3D space. Other known, or yet to be discovered, systematic biases in aftershock detection and identification, such as short-term incompleteness [21,22,23] might also condition parameter estimation.

To keep branching subcritical, exponents α>b are only feasible in subcritical sub-avalanche statistics, i.e., in the presence of the cutoff to the power-law distribution that appear naturally in the formulated BMF model. It is also yet to see the effects of including SOC and facilitation mechanisms to the model, leading to a non-stationary, and potentially transiently supercritical, $\mu_{0}\left(t\right)$ [55]. The emergent transition between SOC and supercritical regimes has been proposed to underlay the observed coexistence or transitions between Gutenberg–Richter and characteristic earthquake regimes [69] and introduce temporal dependencies within the earthquake cycle [66,67]. A temporally dependent $\mu_{h}\left(t\right)$ within the supercritical avalanche, a.k.a. the aftershock tree, can modify as well the α value at the CLT limit. Sub-avalanche propagation in low-dimensional systems exhibit generational memory and do not necessary attain the branching relation in Equation (11). Geometrical constrains might act as an alternative restraining mechanism to tree growth, leading to the compatibility between scale-free sub-avalanche statistics and aftershock production. In all such different scenarios, the emergence of a stable point is foreseeable, which could coincide with the stable scale-free limit assumed in BASS even in the mean-field approximation. Otherwise, the results of this model do not explain the α=b limit of BASS, which appears both in natural catalogs within tectonic settings and acoustic emission experiments on systems encompassing diverse universality classes from mean field to depinning transitions.

The results of this paper may also motivate a reinterpretation of ETAS models within stochastic process frameworks. In the BMF model, the stochastic representation determining the statistics of aftershock production is naturally defined in the space of the ’mean-field’ rather than in the conventional physical ’time’ where the temporal clustering is identified. The renewal interpretation of the statistics in the mean-field space suggests that the productivity, in the terms of the temporal point process in Equation (1), should not be regarded as constant but rather the measure of a finite resource progressively consumed by its direct offspring. In this view, each aftershock simultaneously adds its own triggering to the activity and reduces the remaining triggering potential available to its direct ancestor through a shadowing mechanism associated with the exhaustion of residual stresses. Such a process lies outside the classic self-excitating point process formalism, where each offspring event is generated independently, but can be incorporated as an additional memory effect. Alternative hierarchical descriptions of seismicity based on avalanche-like relaxation cascades and memory effects have also been proposed [67]. Such interpretations are also consistent with broader notions of resource-limited branching dynamics considered in self-similar and stress-constrained extensions of epidemic aftershock models [70,71,72,73]. These approaches generally modify the offspring statistics by introducing dependencies in aftershock sizes while preserving independent production among descendants, which is a mechanism frequently debated in natural seismicity [74,75,76,77]. On the contrary, all aftershocks and root events in the BMF are instead i.i.d. from the same statistical law in Equation (8). Overdispersion in the BMF model is instead attained with the aforementioned dynamical redistribution of triggering capacity, where the production term of the parent decreases in proportion to the aftershock size, moment or energy. The implications may also extend to aftershock identification techniques, many of which rely on ETAS-based metrics to infer causal relationships between events [59].

## 6. Conclusions

We have shown how temporal clustering appears naturally in a mean field model with uncorrelated disorder and two modes of relaxation with different temporal scales, mimicking the dynamics of a general category of potential aftershock production mechanisms. The temporal clustering can be interpreted in terms of aftershock production with unique causal links where each sub-avalanche parent event destabilizes a mean-field interval where aftershocks can initiate. The results obtained in this conceptual model are generalizable to mean-field models defined over a discrete field of random disorder and positive interactions with a temporal profile described in the form Equation (9). This includes multimodal modes combining the different mechanisms described in Section 1, but it might be extensible to avalanche dynamics in other areas exhibiting temporal clustering.

The number of direct aftershocks follows the renewal counting process with refractory field intervals defined in Equation (16). We find an exact solution at the CLT limit, returning Gaussian numbers where the expected value and variance in Equations (19) and (20) scale with the duration of the effective portion of the interval where aftershocks can initiate, with a factor that only depends on the branching parameter of the sub-avalanche propagation ($\mu_{h}$). The mean aftershock production follows a linear law, while the fluctuations exhibit a non-Poisson dependence entirely controlled by $\mu_{h}$. The overdispersion regime, occurring close to the critical limit ($\mu_{h}≲1$), is consistent with field observations, and it appears as a statistical feature even in homogeneous systems rather than owing to the regional or temporal heterogeneity of the underlying geomechanics. Such overdispersion had previously motivated the ad hoc inclusion of geometric or negative-binomial offspring distributions into the ETAS models [27,34,35,37]. The resulting renewal model in the present work provides an indirect way to generate overdispersion based on physical mechanisms, which can be used to guide new stochastic model formulations closer to first principles and physical constrains and reduce systematic biases in parameter estimation and exceedance probabilities, ultimately influencing the assessment of short-term seismic hazard and emergency management following large earthquakes.

The mean-field formulation provides a simplified framework useful to derive stochastic models from first principles but comes with its own limitations. Some specific results, such as the memory on the relation between production and generational-depth, the impossibility of obtaining scale-free aftershock production within finite trees, the high α-values, and the sorted topology of trees are inherent to the mean-field formulation and will foreseeably disappear in low-dimensional systems. Mean-field inconsistencies with real catalogs, as well as a comparative study with operational metric identification techniques, will require examination in finite-dimensional systems such as interface depinning models with viscoelastic relaxation [40], plastic yielding and elastoplastic models [78], spring-block models [39,44,46], or fiber bundle models [79]. The inclusion of SOC and facilitation mechanisms in the mean-field model might lead to transient supercritical regimes with time-dependent parameters and could constrain the α-values below the self-similar limit.

## Data Availability

The data presented in this study are openly available in [Aftershock production in a mean-field avalanche model with bimodal relaxation] at [10.5281/zenodo.21914866].

## References

[1] Sethna J., Dahmen K., Myers C. (2001). Crackling noise. Nature.

[2] Salje E.K., Dahmen K.A. (2014). Crackling noise in disordered materials. Annu. Rev. Condens. Matter Phys..

[3] Lockner D. (1993). The role of acoustic emission in the study of rock fracture. Int. J. Rock Mech. Min. Sci. Geomech. Abstr..

[4] Barés J., Wang D., Wang D., Bertrand T., O’Hern C.S., Behringer R.P. (2017). Local and global avalanches in a two-dimensional sheared granular medium. Phys. Rev. E.

[5] Davidsen J., Goebel T., Kwiatek G., Stanchits S., Baró J., Dresen G. (2021). What Controls the Presence and Characteristics of Aftershocks in Rock Fracture in the Lab?. J. Geophys. Res. Solid Earth.

[6] Li H., Valdés E., Ge Z., Planes A., Jiang X., Huang S., Vives E. (2026). Acoustic Emission Characteristics in Coal Failure from Chinese Coal. Nat. Resour. Res..

[7] Barés J., Dubois A., Hattali L., Dalmas D., Bonamy D. (2018). Aftershock sequences and seismic-like organization of acoustic events produced by a single propagating crack. Nat. Commun..

[8] Chen M., Xiao Y., Jiang X., Wu B., Liu H., Chu J. (2025). Universal avalanches and pressure-dependent scaling law in granular shearing. Acta Geotech..

[9] Baró J., Corral A., Illa X., Planes A., Salje E.K.H., Schranz W., Soto-Parra D.E., Vives E. (2013). Statistical Similarity between the Compression of a Porous Material and Earthquakes. Phys. Rev. Lett..

[10] Nataf G.F., Castillo-Villa P.O., Baró J., Illa X., Vives E., Planes A., Salje E.K. (2014). Avalanches in compressed porous SiO_2_-based materials. Phys. Rev. E.

[11] Baró J., Dahmen K.A., Davidsen J., Planes A., Castillo P.O., Nataf G.F., Salje E.K.H., Vives E. (2018). Experimental Evidence of Accelerated Seismic Release without Critical Failure in Acoustic Emissions of Compressed Nanoporous Materials. Phys. Rev. Lett..

[12] Mäkinen T., Miksic A., Ovaska M., Alava M.J. (2015). Avalanches in Wood Compression. Phys. Rev. Lett..

[13] Viitanen L., Ovaska M., Alava M.J., Karppinen P. (2017). Predicting crackling noise in compressional deformation. J. Stat. Mech. Theory Exp..

[14] Ribeiro H.V., Costa L.S., Alves L.G.A., Santoro P.A., Picoli S., Lenzi E.K., Mendes R.S. (2015). Analogies Between the Cracking Noise of Ethanol-Dampened Charcoal and Earthquakes. Phys. Rev. Lett..

[15] Xu Y., Borrego A.G., Planes A., Ding X., Vives E. (2019). Criticality in failure under compression: Acoustic emission study of coal and charcoal with different microstructures. Phys. Rev. E.

[16] Baró J., Martín-Olalla J.M., Romero F.J., Gallardo M.C., Salje E.K.H., Vives E., Planes A. (2014). Avalanche correlations in the martensitic transition of a Cu–Zn–Al shape memory alloy: Analysis of acoustic emission and calorimetry. J. Phys. Condens. Matter.

[17] Ogata Y. (1988). Statistical Models for Earthquake Occurrences and Residual Analysis for Point Processes. J. Am. Stat. Assoc..

[18] Hawkes A.G., Oakes D. (1974). A cluster process representation of a self-exciting process. J. Appl. Probab..

[19] Utsu T., Ogata Y., Matsu’ura R.S. (1995). The centenary of the Omori formula for a decay law of aftershock activity. J. Phys. Earth.

[20] Ogata Y. (1998). Space-Time Point-Process Models for Earthquake Occurrences. Ann. Inst. Stat. Math..

[21] Lippiello E., Cirillo A., Godano C., Papadimitriou E., Karakostas V. (2019). Post Seismic Catalog Incompleteness and Aftershock Forecasting. Geosciences.

[22] Hainzl S. (2021). ETAS-Approach Accounting for Short-Term Incompleteness of Earthquake Catalogs. Bull. Seismol. Soc. Am..

[23] Mizrahi L., Nandan S., Wiemer S. (2021). Embracing Data Incompleteness for Better Earthquake Forecasting. J. Geophys. Res. Solid Earth.

[24] Utsu T., Seki A. (1955). A Relation between the Area of After-shock Region and the Energy of Main-shock. J. Seismol. Soc. Jpn..

[25] Dascher-Cousineau K., Brodsky E.E., Lay T., Goebel T.H.W. (2020). What Controls Variations in Aftershock Productivity?. J. Geophys. Res. Solid Earth.

[26] Gutenberg B., Richter C.F. (1944). Frequency of earthquakes in California. Bull. Seismol. Soc. Am..

[27] Saichev A., Sornette D. (2004). Anomalous power law distribution of total lifetimes of branching processes: Application to earthquake aftershock sequences. Phys. Rev. E.

[28] Baró J. (2020). Topological Properties of Epidemic Aftershock Processes. J. Geophys. Res. Solid Earth.

[29] Holliday J.R., Turcotte D.L., Rundle J.B., Camacho A.G., Díaz J.I., Fernández J. (2008). A Review of Earthquake Statistics: Fault and Seismicity-Based Models, ETAS and BASS. Earth Sciences and Mathematics: Volume 1.

[30] Lennartz S., Bunde A., Turcotte D.L. (2011). Modelling seismic catalogues by cascade models: Do we need long-term magnitude correlations?. Geophys. J. Int..

[31] Zaliapin I., Ben-Zion Y. (2013). Earthquake clusters in southern California II: Classification and relation to physical properties of the crust. J. Geophys. Res. Solid Earth.

[32] Hainzl S., Brietzke G.B., Zöller G. (2010). Quantitative earthquake forecasts resulting from static stress triggering. J. Geophys. Res. Solid Earth.

[33] Mignan A. (2018). Utsu aftershock productivity law explained from geometric operations on the permanent static stress field of mainshocks. Nonlinear Process. Geophys..

[34] Kagan Y.Y. (2010). Statistical distributions of earthquake numbers: Consequence of branching process. Geophys. J. Int..

[35] Shebalin P.N., Narteau C., Baranov S.V. (2020). Earthquake productivity law. Geophys. J. Int..

[36] Baranov S., Narteau C., Shebalin P. (2022). Modeling and Prediction of Aftershock Activity. Surv. Geophys..

[37] Molchan G., Peresan A. (2024). Number of aftershocks in epidemic-type seismicity models. Geophys. J. Int..

[38] Ben-Zion Y., Lyakhovsky V. (2006). Analysis of aftershocks in a lithospheric model with seismogenic zone governed by damage rheology. Geophys. J. Int..

[39] Hainzl S., Zöller G., Kurths J. (1999). Similar power laws for foreshock and aftershock sequences in a spring-block model for earthquakes. J. Geophys. Res. Solid Earth.

[40] Jagla E.A., Kolton A.B. (2010). A mechanism for spatial and temporal earthquake clustering. J. Geophys. Res. Solid Earth.

[41] Jagla E.A., Landes F.m.c.P., Rosso A. (2014). Viscoelastic Effects in Avalanche Dynamics: A Key to Earthquake Statistics. Phys. Rev. Lett..

[42] Jagla E.A. (2014). Aftershock production rate of driven viscoelastic interfaces. Phys. Rev. E.

[43] Lippiello E., Giacco F., Marzocchi W., Godano C., De Arcangelis L. (2015). Mechanical origin of aftershocks. Sci. Rep..

[44] Zhang X., Shcherbakov R. (2016). Power-law rheology controls aftershock triggering and decay. Sci. Rep..

[45] Baró J., Davidsen J. (2018). Universal avalanche statistics and triggering close to failure in a mean-field model of rheological fracture. Phys. Rev. E.

[46] Motuzas C.A., Shcherbakov R. (2023). Viscoelastic Slider Blocks as a Model for a Seismogenic Fault. Entropy.

[47] Nur A., Booker J.R. (1972). Aftershocks caused by pore fluid flow?. Science.

[48] Rice J.R., Gu J.c. (1983). Earthquake aftereffects and triggered seismic phenomena. Pure Appl. Geophys..

[49] Ross Z.E., Rollins C., Cochran E.S., Hauksson E., Avouac J.P., Ben-Zion Y. (2017). Aftershocks driven by afterslip and fluid pressure sweeping through a fault-fracture mesh. Geophys. Res. Lett..

[50] Helmstetter A., Shaw B.E. (2009). Afterslip and aftershocks in the rate-and-state friction law. J. Geophys. Res. Solid Earth.

[51] Cattania C., Hainzl S., Wang L., Enescu B., Roth F. (2015). Aftershock triggering by postseismic stresses: A study based on Coulomb rate-and-state models. J. Geophys. Res. Solid Earth.

[52] Freed A.M. (2005). Earthquake triggering by static, dynamic, and postseismic stress transfer. Annu. Rev. Earth Planet. Sci..

[53] Ispánovity P.D., Ugi D., Péterffy G., Knapek M., Kalácska S., Tüzes D., Dankházi Z., Máthis K., Chmelík F., Groma I. (2022). Dislocation avalanches are like earthquakes on the micron scale. Nat. Commun..

[54] Baró J., Corral A. (2025). A universal route from avalanches in mean-field models with random fields to stochastic Poisson branching events. Chaos Interdiscip. J. Nonlinear Sci..

[55] Dahmen K.A., Ben-Zion Y., Uhl J.T. (2009). Micromechanical Model for Deformation in Solids with Universal Predictions for Stress-Strain Curves and Slip Avalanches. Phys. Rev. Lett..

[56] Otter R. (1949). The Multiplicative Process. Ann. Math. Stat..

[57] Mainardi F., Spada G. (2011). Creep, relaxation and viscosity properties for basic fractional models in rheology. Eur. Phys. J.-Spec. Top..

[58] Gut A. (2009). Limit Theorems for Stopped Random Walks. Stopped Random Walks: Limit Theorems and Applications.

[59] Baiesi M., Paczuski M. (2004). Scale-free networks of earthquakes and aftershocks. Phys. Rev. E.

[60] Zaliapin I., Gabrielov A., Keilis-Borok V., Wong H. (2008). Clustering Analysis of Seismicity and Aftershock Identification. Phys. Rev. Lett..

[61] Schorlemmer D., Wiemer S., Wyss M. (2005). Variations in earthquake-size distribution across different stress regimes. Nature.

[62] Scholz C.H. (2015). On the stress dependence of the earthquake b value. Geophys. Res. Lett..

[63] Wiemer S., Wyss M. (2002). Mapping spatial variability of the frequency-magnitude distribution of earthquakes. Advances in Geophysics.

[64] Zaliapin I., Ben-Zion Y. (2013). Earthquake clusters in southern California I: Identification and stability. J. Geophys. Res. Solid Earth.

[65] Main I. (1996). Statistical physics, seismogenesis, and seismic hazard. Rev. Geophys..

[66] Tormann T., Enescu B., Woessner J., Wiemer S. (2015). Randomness of megathrust earthquakes implied by rapid stress recovery after the Japan earthquake. Nat. Geosci..

[67] Rodkin M. (2019). Application of the multiplicative cascade model for the description of the seismic regime and for the seismic hazard assessment. IOP Conf. Ser. Earth Environ. Sci..

[68] Hanks T.C., Hiroo K. (1979). A moment magnitude scale. J. Geophys. Res. Solid Earth.

[69] Dahmen K., Ertaş D., Ben-Zion Y. (1998). Gutenberg-Richter and characteristic earthquake behavior in simple mean-field models of heterogeneous faults. Phys. Rev. E.

[70] Saichev A., Sornette D. (2005). Vere-Jones’ self-similar branching model. Phys. Rev. E.

[71] Turcotte D.L., Holliday J.R., Rundle J.B. (2007). BASS, an alternative to ETAS. Geophys. Res. Lett..

[72] Rundle J.B., Turcotte D.L., Holliday J.R., Van Aalsburg J. (2008). A stress dependent BASS model for earthquakes, damage and fracture. Proceedings of the Geophysical Research Abstracts.

[73] Petrillo G., Lippiello E., Zhuang J. (2024). Including stress relaxation in point-process model for seismic occurrence. Geophys. J. Int..

[74] Lippiello E., de Arcangelis L., Godano C. (2008). Influence of Time and Space Correlations on Earthquake Magnitude. Phys. Rev. Lett..

[75] Davidsen J., Green A. (2011). Are Earthquake Magnitudes Clustered?. Phys. Rev. Lett..

[76] Lippiello E., Godano C., de Arcangelis L. (2012). The earthquake magnitude is influenced by previous seismicity. Geophys. Res. Lett..

[77] Grimm C., Hainzl S., Käser M., Küchenhoff H. (2022). Solving three major biases of the ETAS model to improve forecasts of the 2019 Ridgecrest sequence. Stoch. Environ. Res. Risk Assess..

[78] Nicolas A., Ferrero E.E., Martens K., Barrat J.L. (2018). Deformation and flow of amorphous solids: Insights from elastoplastic models. Rev. Mod. Phys..

[79] Lennartz-Sassinek S., Main I.G., Danku Z., Kun F. (2013). Time evolution of damage due to environmentally assisted aging in a fiber bundle model. Phys. Rev. E.

